# Self-reported hepatitis A and B vaccination coverage among men who have sex with men (MSM), associated factors and vaccination recommendations in 43 countries of the WHO European Region: results from the European MSM Internet Survey, EMIS-2017

**DOI:** 10.2807/1560-7917.ES.2024.29.45.2400100

**Published:** 2024-11-07

**Authors:** Michael Brandl, Axel J Schmidt, Ulrich Marcus, Erika Duffell, Ettore Severi, Antons Mozalevskis, Anda Kivite-Urtane, Matthias an der Heiden, Sandra Dudareva

**Affiliations:** 1 Department of Infectious Disease Epidemiology, Robert Koch Institute (RKI), Berlin, Germany; 2 Charité – Universitätsmedizin Berlin, corporate member of Freie Universität Berlin and Humboldt-Universität zu Berlin, Berlin, Germany; 3 Sigma Research, Department of Public Health, Environments and Society, London School of Hygiene and Tropical Medicine (LSHTM), London, United Kingdom; 4 European Centre for Disease Prevention and Control (ECDC), Stockholm, Sweden; 5 World Health Organization (WHO) Regional Office for Europe, Copenhagen, Denmark; 6 Institute of Public Health, Riga Stradins University, Riga, Latvia

**Keywords:** hepatitis A, hepatitis B, vaccination coverage, Europe, men who have sex with men

## Abstract

**Background:**

Hepatitis A and B vaccinations are recommended for men who have sex with men (MSM), given their increased risk of infection. However, data on vaccination programmes are scarce.

**Aim:**

To use information on vaccination recommendations and vaccine uptake among MSM in the WHO European Region to guide prevention.

**Methods:**

From a large pan-European MSM Internet Survey (EMIS-2017), we analysed data on self-reported hepatitis A and B vaccination status by age, education, financial coping, settlement size, outness (disclosure of sexual behaviour), migration history and diagnosis with hepatitis C or HIV, using multivariable logistic regression. Additionally, we collected information on national hepatitis A and B vaccination recommendations.

**Results:**

We present data of 113,884 MSM, median age 36 years (IQR: 27–47). Vaccination for hepatitis A and B was recommended and free for MSM in 7 and 18 of 43 countries, respectively. Of all respondents, 48% (n = 50,966) reported ever being vaccinated against hepatitis A, and 53% (n = 56,889) against hepatitis B. Odds for being vaccinated against hepatitis A increased with outness (‘out to (almost) all’ aOR: 1.78, 95% CI: 1.72–1.85 vs ‘out to none’) and were higher in countries where vaccination was recommended and free for MSM (aOR: 2.22, 95% CI: 1.29–3.82 vs ‘no recommendation’). Results for hepatitis B were similar (outness: aOR: 1.81, 95% CI: 1.75–1.88 and MSM-specific vaccination recommendation: aOR: 2.44, 95% CI: 1.54–3.85).

**Conclusion:**

Large proportions of MSM in Europe remain vulnerable to hepatitis A and B, despite available vaccination. Implementation of MSM-specific vaccination recommendations and greater efforts to improve the societal climate for MSM are needed to address gaps in vaccine coverage.

**Key public health message**

**What did you want to address in this study and why?**

Safe and effective vaccines against hepatitis A and B have long been available and doctors recommend that men who have sex with men (MSM) are vaccinated. Still, vaccination coverage among MSM in the WHO European Region is insufficient and hepatitis A outbreaks and hepatitis B infections occur. We aimed to address hepatitis A and B vaccine uptake, associated factors and national vaccination recommendations among MSM.

**What have we learnt from this study?**

In 2017–18, only about one in two MSM in the WHO European Region were vaccinated against hepatitis A and B, with large variability across the 43 investigated countries. In countries where vaccination was recommended in national guidelines and where men could be open about their sexual orientation, men were more likely to be protected from hepatitis infections through being vaccinated.

**What are the implications of your findings for public health?**

MSM in Europe are still vulnerable to hepatitis A and B virus infections. Inclusion of MSM in national vaccination guidelines is important but not sufficient to close vaccination gaps. Active recommendation and coverage of all or parts of the vaccination costs increase chances for MSM to get vaccinated. Reducing stigma and creating an open environment that enables MSM to follow recommendations will be crucial on the path to hepatitis elimination.

## Introduction

The hepatitis A virus causes an acute liver infection and is transmitted through oral contact with faecal matter, with outbreaks frequently caused by contaminated food [1]. In most countries of the World Health Organization (WHO) European Region, hepatitis A prevalence is low, but outbreaks occur. Therefore, vaccination is recommended in endemic areas, outbreak situations and for specific groups considered to be at higher risk of infection, such as men who have sex with men (MSM) [2,3]. From 2016 to 2018, outbreaks among MSM attributed to sexual contact were reported in multiple countries of the WHO European Region with at least 1,400 confirmed cases, of which more than half required hospitalisation [4].

Infections with hepatitis B virus can also cause acute disease or, especially in children, stay asymptomatic and become chronic, potentially leading to long-term complications like liver cirrhosis and hepatocellular carcinoma [5,6]. Sexual transmission among MSM was the second most common route for acute infections reported in the European Union/European Economic Area (EU/EEA) in 2022 [7]. With no curative therapy available and lifelong medication as the only treatment option for the prevention of long-term complications, immunisation remains the cornerstone of hepatitis B prevention. The implementation of national hepatitis B vaccination programmes led to major reductions in hepatitis B incidence across the WHO European Region over recent years, but adults born before the introduction of universal hepatitis B vaccination remain at risk of infection [8,9].

Vaccines against both hepatitis A and B were developed in the 1980s and became widely available in the 1990s [10,11]. In the Action plan for the health sector response to viral hepatitis in the WHO European Region, WHO set the target to develop and implement national vaccination guidelines for groups at risk, including MSM [12]. However, data on vaccination programmes and vaccine coverage among MSM in Europe are scarce and research on hepatitis B in the European MSM Internet Survey 2010 (EMIS-2010) was one of the only available sources [13]. Previous evidence from analysis of EMIS-2010 suggested that uptake of hepatitis B vaccination and other MSM health services is positively associated with outness, the disclosure of one's sexual behaviour and identity [13-15].

The WHO identified MSM as one of five key populations for hepatitis elimination and recommended that hepatitis B vaccination should be included as part of the package of essential health services [16]. In 2024, the Council of the European Union highlighted the need for monitoring of vaccination coverage among MSM and other key population groups for hepatitis B [17]. Recent hepatitis A outbreaks in the WHO European Region among MSM may have influenced vaccine uptake and led to changes in vaccination programmes. For this study, we collected information on hepatitis A and B vaccination recommendations and analysed vaccine uptake and associated factors among MSM. We used data from a survey undertaken in 50 predominantly European countries in 2017–18 to guide the effective targeting of prevention measures. Our results provide an updated overview of the progress in hepatitis A and B vaccination among MSM and help monitoring elimination efforts in the WHO European Region. 

## Methods

### Data sources

#### EMIS-2017

We used data from the European MSM Internet Survey 2017 (EMIS-2017), an open access internet survey that collected data on sexual health from 18 October 2017 to 31 January 2018 with a total of 127,792 eligible respondents from 50 countries. Detailed methodology of EMIS-2017 and a full list of countries have been described elsewhere [18,19]. Survey participants were above the age of sexual consent in their country, identified as men or transgender men and indicated that they either were sexually attracted to men and/or have had sex with men. We excluded participants who answered inconsistently on questions regarding age (seven possible inconsistencies), steady male partners (five possible inconsistencies) and non-steady partners (six possible inconsistencies), as outlined in the EMIS-2017 report [18]. We also excluded participants who did not answer the two hepatitis vaccination questions. 

For this study, we included only participants from countries within the WHO European Region with a minimum of 100 participants. Participants from four European microstates with lower case numbers were included in the datasets of neighbouring countries: Andorra in Spain, Liechtenstein in Switzerland, Monaco in France and San Marino in Italy [19]. Participants from the neighbouring Western Balkan countries Albania, Kosovo* and Montenegro were grouped together to exceed this threshold, as described in the EMIS-2017 report [18]. All participants gave informed consent.

#### Vaccination recommendations

We collected information on national vaccination recommendations for MSM for hepatitis A and B from each included country. For this, we created two short surveys including three questions: (i) if MSM were included in hepatitis A or B vaccination recommendations, (ii) when the recommendation was introduced, and (iii) how costs for vaccines were covered. The questionnaires also provided the opportunity to share any additional information on the national hepatitis vaccination programmes. In 2020/21, we sent the two surveys for hepatitis A and B out to European Centre for Disease Prevention and Control (ECDC) and WHO national focal points for hepatitis. In case of no response, we contacted public health specialists and other national experts with access to these data in their respective countries based on recommendations from established European and global public health networks. We also collected information on existing national hepatitis A and B vaccination programmes for infants and children from an online search and literature review [20,21].

### Variables

#### EMIS-2017

For the analysis of the self-reported hepatitis A and B vaccination status, we created two binary variables for vaccination history by coding the answers to the two questions ‘Have you been vaccinated against hepatitis A?’ and ‘Have you been vaccinated against hepatitis B?’ as either vaccinated or not vaccinated. Participants with a full or partial vaccination history for hepatitis A and B, respectively, responded either ‘Yes, and I completed the course’, ‘Yes, but I did not complete the course’, or ‘Yes, but I did not respond to the vaccinations’ (only hepatitis B). Non-response to vaccination is defined as low antibody levels after hepatitis B vaccination, but no further explanation was provided to participants while completing the EMIS-2017 questionnaire. No vaccination history included the answers ‘No, and I don't know if I'm immune’ or ‘I don't know’. We excluded participants who answered ‘No, because I've had hepatitis A/B (and am now naturally immune)’ or ‘No, I have chronic hepatitis B infection’ (only hepatitis B).

We also used one of five questions on viral hepatitis knowledge and immunisation awareness among MSM in Europe [22]. We looked at the statement ‘Doctors recommend MSM are vaccinated against both hepatitis A and B’ and coded participants who answered ‘I knew this already’ as ‘aware of recommendation’ and participants who answered differently (‘I wasn’t sure about this’, ‘I didn’t know this already’, ‘I don’t understand this’, ‘I do not believe this’) as ‘not aware’.

From EMIS-2017, we additionally used the following categorical variables: age in years (< 25; 25–39; ≥ 40), settlement size according to numbers of inhabitants (medium-sized or smaller settlements: < 500,000; big to very big cities: ≥ 500,000), education according to years spent in full time education beyond the age of 16 years (low: 0–1 year; mid: at least upper secondary, 2–5 years; high: first stage of tertiary or more, ≥ 6 years), financial coping described as feelings about participants' income (struggling/really struggling with present income, neither comfortable nor struggling with present income, living comfortably/really comfortably on present income), outness defined as the proportion of people in participants' life (family, friends, and work or study colleagues) that know about their sexual attraction to men (out to none or few, out to some, out to all or almost all), and infection status for hepatitis C virus (HCV) and human immunodeficiency virus (HIV) (ever diagnosed with HCV or HIV; never diagnosed with either HCV or HIV).

To assess migration history, participants stated if they were born outside their current country of residence and we grouped their countries of birth into five categories: (i) EU/EEA, Switzerland and the United Kingdom (UK); (ii) other countries of the WHO European Region including Central Asian, Eastern European and Western Balkan countries, Israel and Türkiye; (iii) WHO Eastern Mediterranean Region; (iv) Australia, Canada, New Zealand or United States (US); (v) all other countries.

#### Vaccination recommendations

From the results of the focal point surveys, we created two variables regarding MSM-specific vaccination recommendations for hepatitis A and B with four values (no recommendation; recommendation with full out-of-pocket payment, i.e. that the recipient covers the full vaccination costs; recommendation with co-payment, i.e. that the recipient needs to cover only a portion of the vaccination costs; recommendation with free of charge vaccination). All participants in EMIS-2017 from a specific country were then assigned to the respective value. For analysis, we used recommendations that were implemented before the year 2018, when EMIS-2017 was available online, and reported later introductions separately. Where possible, the results were cross-checked with available literature [23-26].

For variable universal hepatitis B vaccination programmes, we used the year of introduction of the programme and the reached age groups according to results from the literature review [21,27,28]. We identified participants in the EMIS-2017 dataset who were born in years in which national universal hepatitis B vaccination programmes had already been implemented and created two values (not reached; age groups potentially reached).

### Statistical analysis

#### Description

We described participants with absolute numbers and proportions by age, settlement size, education, financial coping, outness, HCV/HIV status and migration history. Additionally, we reported how many participants lived in countries with MSM-specific vaccination recommendations for hepatitis A and B and were potentially reached by universal hepatitis B vaccination programmes. We stratified results by the outcomes hepatitis A and B vaccination history. Participants with missing values in relevant variables were excluded from calculations of proportions but we reported their absolute numbers. Additionally, we reported numbers and proportions of participants who stated they were not vaccinated and of participants who did not know their vaccination status for variables described above. 

We calculated proportions of participants with hepatitis A and B vaccination history by country of residency and displayed national results on maps of Europe. Maps were created using the ECDC Map Maker tool (EMMa; https://geoportal.ecdc.europa.eu/mapmaker).

#### Uni- and multivariable analysis

In univariable analysis, we used logistic regression with the outcomes of hepatitis A and B vaccination history and the variables described above. We reported odds ratios (OR) and 95% confidence intervals (95% CI). To adjust for potential confounding, we used multivariable logistic regression analysis reporting adjusted OR (aOR) and 95% CI and added variables from univariable analysis into a forward selection process. In the final models, we included age, settlement size, education, financial coping, outness, HCV/HIV diagnosis, country of birth, MSM-specific hepatitis A/B vaccination recommendation and universal hepatitis B vaccination programme (only hepatitis B). We applied a multilevel design with the two levels, participants and countries, using countries as random intercept and reported the intraclass correlation coefficient (ICC). In sensitivity analyses, we ran these multivariable, multilevel models and excluded participants who either did not know their vaccination status or who may have been reached by universal hepatitis B vaccination programmes.

#### Correlation

We used Pearson correlation at country level to analyse the association between outness and hepatitis A and B vaccination history. For each country, we calculated the proportion of participants who were out to (almost) all of the people they knew and the proportion who had a hepatitis A and B vaccination history, respectively. In two scatter plots, we displayed the results for all countries with regression lines and 95% CI and reported correlation coefficients R with p values. We included the results of the short focal point surveys on vaccination recommendations for MSM in the figure as colours for country groups.

Additionally, we analysed the correlation between the proportion of participants per country who were aware that doctors generally recommend hepatitis vaccination for MSM and, where this information was available, the time when an actual national MSM-specific vaccination recommendation for hepatitis A and B was introduced.

## Results

The analytic sample included 113,884 participants from 43 countries in the WHO European Region. The median age of participants was 36 years (IQR: 27–47). Regarding hepatitis A vaccination, 45,745 (40%) reported a full vaccination course, 5,221 (5%) a partial vaccination course, 30,092 (26%) no vaccination or awareness of immunity through previous infection and 24,197 (21%) that they did not know. We excluded 8,048 (7%) who had hepatitis A in the past and were immune, and 581 (0.5%) participants who did not answer the question. Regarding hepatitis B vaccination, 51,203 (45%) reported full vaccination, 4,318 (4%) partial vaccination, 1,368 (1%) non-response to vaccination, 26,294 (23%) no vaccination and no awareness of immunity, and 23,370 (21%) that they did not know. We excluded 6,318 (6%) participants who were naturally immune and 485 (0.4%) chronically infected, as well as 528 (0.5%) who did not answer the question. This resulted in 50,966 (48%) of 105,255 eligible participants reporting hepatitis A vaccination history and 56,889 (53%) of 106,553 eligible participants reporting hepatitis B vaccination history. Among the 101,724 eligible participants with information for hepatitis A and B vaccination, 45,197 (44%) reported to have received both vaccinations. Detailed characteristics of participants are displayed in Table 1.

**Table 1:** Description of MSM study sample by hepatitis A (n = 105,255) and B vaccination history (n = 106,553) in 43 WHO European Region countries, EMIS-2017

Variables	Hepatitis A vaccination history					Hepatitis B vaccination history				
	No n = 54,289		Yes n = 50,966		Total n = 105,255	No n = 49,664		Yes n = 56,889		Total n = 106,553
	n	%	n	%	n	n	%	n	%	n
**Age group in years**										
< 25	13,413	65	7,070	35	20,483	12,703	62	7,883	38	20,586
25–39	23,613	50	23,367	50	46,980	21,017	44	26,701	56	47,718
≥ 40	17,263	46	20,529	54	37,792	15,944	42	22,305	58	38,249
**Settlement size**										
Medium-sized or smaller (< 500,000 inhabitants)	31,144	54	26,578	46	57,722	28,524	49	29,764	51	58,288
Big to very big cities (≥ 500,000 inhabitants)	22,542	49	23,895	51	46,437	20,574	44	26,579	56	47,153
Missing	603		493		1,096	566		546		1,112
**Education**										
Low (0–1 year post age 16 years)	2,945	62	1,792	38	4,737	2,723	58	2,005	42	4,728
Mid (at least upper secondary; 2–5 years post age 16 years)	19,935	55	16,040	45	35,975	18,887	52	17,294	48	36,181
High (first stage of tertiary or more; ≥ 6 years post age 16 years)	27,500	48	29,939	52	57,439	24,424	42	34,086	58	58,510
Missing	3,909		3,195		7,104	3,630		3,504		7,134
**Financial coping**										
Struggling/really struggling with present income	10,533	59	7,251	41	17,784	9,690	54	8,165	46	17,855
Neither comfortable nor struggling with present income	20,389	57	15,259	43	35,648	18,684	52	17,396	48	36,080
Living comfortably/really comfortably with present income	23,109	45	28,308	55	51,417	21,037	40	31,177	60	52,214
Missing	258		148		406	253		151		404
**Outness**										
Out to none or few	20,039	65	10,735	35	30,774	18,922	61	12,320	39	31,242
Out to some	15,890	53	13,982	47	29,872	14,247	47	16,045	53	30,292
Out to (almost) all	17,560	41	25,789	59	43,349	15,722	36	28,043	64	43,765
Missing	800		460		1,260	773		481		1,254
**Ever diagnosed with hepatitis C or HIV**										
No	50,856	54	43,301	46	94,157	46,801	49	48,577	51	95,378
Yes	3,151	30	7,346	70	10,497	2,603	25	7,969	75	10,572
Missing	282		319		601	260		343		603
**Country of birth**										
No migration history	48,007	52	43,447	48	91,454	43,898	47	48,577	53	92,475
EU/EEA, Switzerland or UK	3,021	42	4,251	58	7,272	2,712	37	4,636	63	7,348
Other countries of the WHO European Region	1,095	66	557	34	1,652	1,040	62	644	38	1,684
WHO Eastern Mediterranean Region	335	59	232	41	567	292	49	302	51	594
Australia, Canada, New Zealand or US	260	30	597	70	857	234	27	627	73	861
All other countries	1,423	45	1,760	55	3,183	1,343	41	1,968	59	3,311
Missing	148		122		270	145		135		280
**MSM-specific hepatitis A recommendation**										
No recommendation	17,807	66	9,156	34	26,963	NA				
Out-of-pocket	1,961	51	1,858	49	3,819					
Co-payment	10,742	47	12,309	53	23,051					
Free of charge	23,779	46	27,643	54	51,422					
**MSM-specific hepatitis B recommendation**										
No recommendation	NA					9,148	72	3,581	28	12,729
Out-of-pocket						4,631	53	4,159	47	8,790
Co-payment						8,659	42	12,023	58	20,682
Free of charge						27,140	42	37,088	58	64,228
Missing						86		38		124
**Universal hepatitis B programme**										
Not reached	NA					36,902	44	46,185	56	83,087
Age groups potentially reached						12,762	54	10,704	46	23,466

EU/EEA: European Union/European Economic Area; EMIS-2017: European MSM Internet Survey; HIV: human immunodeficiency virus: MSM: men who have sex with men; NA: not applicable; UK: United Kingdom; US: United States; WHO: World Health Organization.

**Figure 1:**
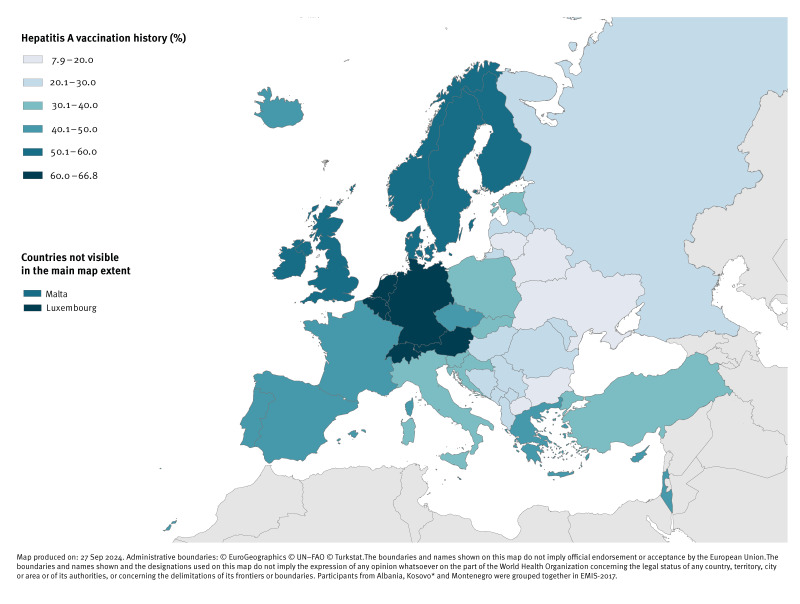
Proportion of participants who ever received a hepatitis A vaccination by country of residency in 43 WHO European Region countries, EMIS-2017 (n = 105,255)

EMIS: European MSM Internet Survey; WHO: World Health Organization.

The highest proportions for hepatitis A vaccination history were reported from participants in Switzerland (67%), Austria (66%), Germany (65%), Luxembourg (65%), the Netherlands (65%), and Belgium (60%); the lowest proportions were reported in Ukraine (8%), North Macedonia (10%), Belarus (13%), Bulgaria (16%) and Lithuania (20%) (Figure 1). Numbers and percentages of participants with vaccination history by country of residency are available in Supplementary Table S1.

* This designation is without prejudice to positions on status and is in line with United Nations Security Council Resolution 1244/99 and the International Court of Justice Opinion on the Kosovo Declaration of Independence.

**Figure 2:**
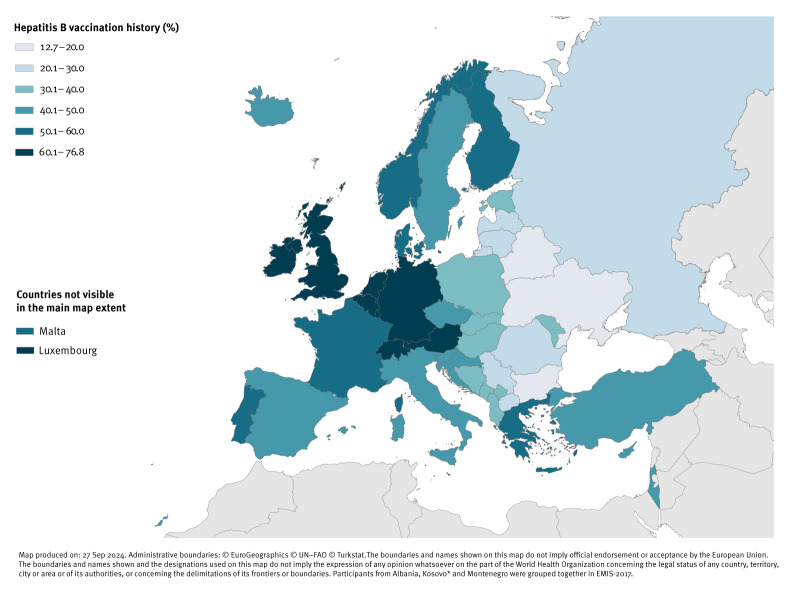
Proportion of participants who ever received a hepatitis B vaccination by country of residency in 43 WHO European Region countries, EMIS-2017 (n = 106,553)

EMIS: European MSM Internet Survey; WHO: World Health Organization.

For hepatitis B, the highest proportion of participants reporting vaccination were found in the Netherlands (77%), Switzerland (70%), Luxembourg (68%), Austria (66%), UK (65%), Germany (65%), and Belgium (64%); the lowest proportions were reported in Ukraine (13%), Belarus (14%) and Bulgaria (19%) (Figure 2).

* This designation is without prejudice to positions on status and is in line with United Nations Security Council Resolution 1244/99 and the International Court of Justice Opinion on the Kosovo Declaration of Independence.

We compared participants who answered that they were not vaccinated against hepatitis A and B with those who did not know their vaccination status and found that higher proportions of participants younger than 25 years and those potentially covered by universal hepatitis B programmes reported not knowing their vaccination status. The results of this descriptive comparison are provided in Supplementary Table S2.

### Uni- and multivariable analysis

In the multivariable analysis with outcome hepatitis A vaccination history, participants under 25 years of age had 28% lower odds of being vaccinated than participants 25–39 years old (Table 2). Participants in big cities were 1.35 times more likely to be vaccinated than participants in smaller settlements and participants with high education were 1.72 times more likely to be vaccinated than participants with low education. Living comfortably to very comfortably increased chances to be vaccinated by 68% compared with struggling on income and being out to (almost) all of friends and family increased chances by 78% compared with being out to no one or only few people. Participants with diagnosis of HCV and/or HIV in the past were 2.75 times more likely to have received hepatitis A vaccination than participants without HCV/HIV diagnosis. Migration history from the WHO European Region outside EU/EEA, Switzerland or the UK was associated with 31% lower odds of being vaccinated, and from the WHO Eastern Mediterranean Region with 32% lower odds, whereas migration history from Australia, Canada, New Zealand or the US was associated with 55% higher odds of being vaccinated. Participants who lived in countries with MSM-specific hepatitis A vaccination recommendations had higher odds of being vaccinated compared with participants from countries without recommendations. Chances of being vaccinated were more than doubled when vaccinations were fully (aOR: 2.22, 95% CI: 1.29–3.82) or partially (aOR: 2.35, 95% CI: 1.37–4.02) covered, but were not increased for participants in countries with out-of-pocket payments (aOR: 1.14, 95% CI: 0.61–2.14). The ICC was 0.11, i.e. 11% of the total variability of the model were explained by differences between countries.

**Table 2:** Univariable and multivariable multilevel analysis for outcome hepatitis A (n = 95,209) and B vaccination history (n = 96,336), 43 WHO European Region countries, EMIS-2017

Variables	Hepatitis A vaccination history				Hepatitis B vaccination history			
	Univariable analysis		Multivariable analysis		Univariable analysis		Multivariable analysis	
	OR	95% CI	aOR	95% CI	OR	95% CI	aOR	95% CI
**Age in years**								
< 25	0.53	0.51–0.55	0.72	0.69–0.75	0.49	0.47–0.51	0.66	0.64–0.69
25–39	Ref.		Ref.		Ref.		Ref.	
≥ 40	1.20	1.17–1.23	0.96	0.93–1.00	1.10	1.07–1.13	0.89	0.86–0.92
**Settlement size**								
Medium-sized or smaller (< 500 000 inhabitants)	Ref.		Ref.		Ref.		Ref.	
Big to very big cities (≥ 500 000 inhabitants)	1.24	1.21–1.27	1.35	1.31–1.39	1.24	1.21–1.27	1.31	1.27–1.35
**Education**								
Low (0–1 year post age 16 years)	Ref.		Ref.		Ref.		Ref.	
Mid (at least upper secondary; 2–5 years post age 16 years)	1.32	1.24–1.41	1.33	1.24–1.42	1.24	1.17–1.32	1.31	1.23–1.41
High (first stage of tertiary or more; ≥ 6 years post age 16 years)	1.79	1.68–1.90	1.72	1.60–1.84	1.90	1.79–2.01	1.92	1.79–2.05
**Financial coping**								
Struggling/really struggling with present income	Ref.		Ref.		Ref.		Ref.	
Neither comfortable nor struggling with present income	1.09	1.05–1.13	1.20	1.15–1.25	1.10	1.07–1.15	1.20	1.15–1.25
Living comfortably/really comfortably with present income	1.78	1.72–1.84	1.68	1.61–1.75	1.76	1.70–1.82	1.64	1.57–1.70
**Outness**								
Out to none or few	Ref.		Ref.		Ref.		Ref.	
Out to some	1.64	1.59–1.70	1.36	1.31–1.41	1.73	1.68–1.79	1.43	1.38–1.49
Out to (almost) all	2.74	2.66–2.83	1.78	1.72–1.85	2.74	2.66–2.82	1.81	1.75–1.88
**Ever diagnosed with hepatitis C or HIV**								
No	Ref.		Ref.		Ref.		Ref.	
Yes	2.74	2.62–2.86	2.75	2.62–2.89	2.95	2.82–3.09	2.96	2.81–3.12
**Country of birth**								
No migration history	Ref.		Ref.		Ref.		Ref.	
EU/EEA, Switzerland or UK	1.55	1.48–1.63	1.05	0.99–1.10	1.54	1.47–1.62	1.04	0.98–1.10
Other countries of the WHO European Region	0.56	0.51–0.62	0.69	0.61–0.78	0.56	0.51–0.62	0.72	0.64–0.81
WHO Eastern Mediterranean Region	0.77	0.65–0.90	0.68	0.56–0.82	0.93	0.80–1.10	0.79	0.66–0.95
Australia, Canada, New Zealand or US	2.54	2.19–2.94	1.55	1.31–1.82	2.42	2.09–2.82	1.40	1.18–1.66
All other countries	1.37	1.27–1.47	1.12	1.03–1.21	1.32	1.23–1.42	1.02	0.94–1.10
**MSM-specific hepatitis A vaccination recommendation**								
No recommendation	Ref.		Ref.		NA			
Out-of-pocket	1.84	1.72–1.97	1.14	0.61–2.14				
Co-payment	2.23	2.15–2.31	2.35	1.37–4.02				
Free of charge	2.26	2.19–2.33	2.22	1.29–3.82				
**MSM-specific hepatitis B vaccination recommendation**								
No recommendation	NA				Ref.		Ref.	
Out-of-pocket					2.29	2.17–2.43	1.62	0.94–2.79
Co-payment					3.55	3.38–3.72	2.95	1.65–5.27
Free of charge					3.49	3.35–3.64	2.44	1.54–3.85
**Universal hepatitis B vaccination programme**								
Not reached	NA				Ref.		Ref.	
Age groups potentially reached					0.67	0.65–0.69	1.06	1.01–1.11

aOR: adjusted odds ratio; CI: confidence interval; EU/EEA: European Union/European Economic Area; HIV: human immunodeficiency virus; MSM: men who have sex with men; NA: not applicable; Ref.: reference; UK: United Kingdom; US: United States; WHO: World Health Organization.

Uni- and multivariable analysis of hepatitis B vaccination showed associations of similar magnitudes for settlement size, education, financial coping, outness, HCV/HIV diagnosis and migration history as analysis of hepatitis A vaccination. The youngest age group of < 25 years had 34% lower chances of being vaccinated against hepatitis B and the oldest age group of ≥ 40-year-old participants had 11% lower chances to be vaccinated than the reference group of 25–39-year-olds in multivariable analysis. We found highest aOR with two to three times higher chances of being vaccinated against hepatitis B among participants living in countries with vaccination recommendations with co- (aOR: 2.95, 95% CI: 1.65–5.27) or full (aOR: 2.44, 95% CI: 1.54–3.85) payment and only a small to no effect for full out-of-pocket payments (aOR: 1.62, 95% CI: 0.94–2.79). The odds of being vaccinated for participants in age groups potentially reached by universal vaccination programmes did not differ much from participants in age groups not reached (aOR: 1.06, 95% CI: 1.01–1.11). The ICC was 0.09 and thus of similar magnitude as for hepatitis A vaccination.

In sensitivity analyses, we excluded participants who did not know their vaccination status (provided in Supplementary Table S3), and participants in age groups potentially reached by universal programmes in childhood (provided in Supplementary Table S4), which yielded results of similar magnitude to the original multivariable regression.

### Vaccination recommendations and correlation between outness and vaccination history

**Figure 3:**
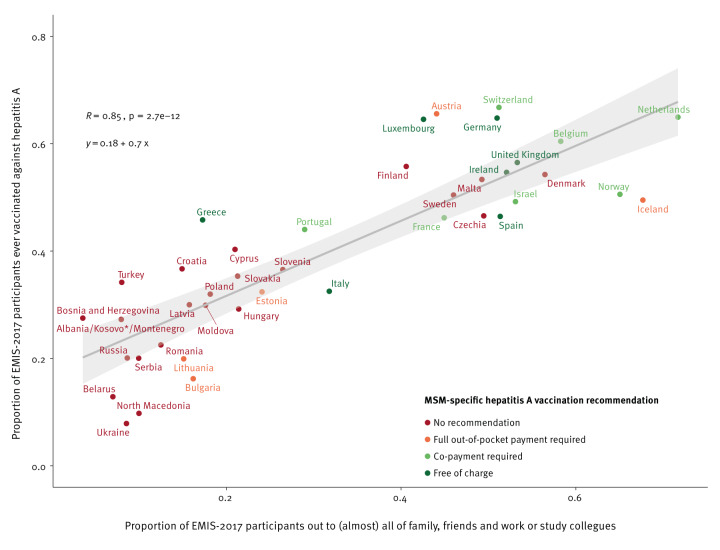
Correlation between outness and hepatitis A vaccination history, including MSM-specific hepatitis A vaccination recommendation, 43 WHO European Region countries, EMIS-2017

EMIS: European MSM Internet Survey; MSM: men who have sex with men; WHO: World Health Organization.

In 19 of 43 countries, hepatitis A vaccination was recommended for MSM; in seven countries, vaccination was free of charge for MSM, in another seven countries it came with a co-payment, and in five countries vaccination had to be paid out-of-pocket. Across countries, outness correlated highly positively with hepatitis A vaccination coverage with a correlation coefficient R of 0.85 and p value < 0.001 (Figure 3). Participants from countries with free and co-pay MSM-specific hepatitis A vaccination recommendations in place were generally more out and had higher proportions of hepatitis A vaccination history. In Italy and Spain, vaccination coverage was lower compared to other countries where MSM had similar outness levels. Participants from Greece and Italy reported lowest levels of outness among countries where vaccination was free for MSM. In countries without MSM-specific recommendations, participants were more likely to be less out and have lower proportions hepatitis A vaccination, with the exceptions of Czechia, Denmark, Finland, Malta and Sweden.

* This designation is without prejudice to positions on status and is in line with United Nations Security Council Resolution 1244/99 and the International Court of Justice Opinion on the Kosovo Declaration of Independence.

**Figure 4:**
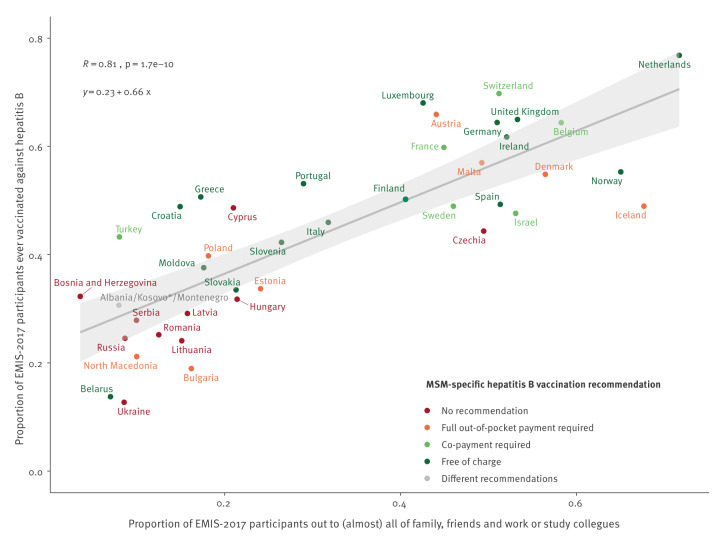
Correlation between outness and hepatitis B vaccination history, including MSM-specific hepatitis B vaccination recommendation, 43 WHO European Region countries, EMIS-2017

EMIS: European MSM Internet Survey; MSM: men who have sex with men; WHO: World Health Organization.

Hepatitis B vaccination was recommended for MSM in 32 of 43 countries; in 18 countries free of charge, in six countries with co-payment and in eight countries with out-of-pocket payment. Outness and hepatitis B vaccination correlated highly positively with an R of 0.81 and p value < 0.001 (Figure 4). The majority of the countries with free-of-charge MSM vaccination in place had above-average proportions of vaccinated participants. In Belarus, Norway, Slovakia, and Spain, the proportions with vaccination were lower than in other countries with free vaccination and similar levels of outness. Eleven countries had no hepatitis B vaccination recommendations at the time of EMIS-2017 and participants from most of them had both low proportions of outness and vaccination.

* This designation is without prejudice to positions on status and is in line with United Nations Security Council Resolution 1244/99 and the International Court of Justice Opinion on the Kosovo Declaration of Independence.

Overall, 59% of participants stated that they knew that doctors generally recommend vaccination for MSM against hepatitis A and B. This proportion was highest among participants from the Netherlands (83%) and lowest among participants from Moldova (28%). The year of introduction of the MSM-specific recommendations was available for 11 countries for hepatitis A and for 26 countries for hepatitis B. The longest period since hepatitis A vaccination has been recommended for MSM was reported by the UK (20 years), and for hepatitis B vaccination by Austria and Norway (32 years). The results of the correlation between year of introduction of hepatitis A and B vaccination recommendation and awareness of the fact that doctors generally recommend vaccination for MSM are provided in Supplementary Figure S1 and Figure S2, respectively. For hepatitis A, the time since vaccination implementation did not correlate (R = 0.18, p = 0.59) with the awareness about the statement that doctors recommended hepatitis vaccination for MSM, and for hepatitis B, we observed a low positive correlation (R = 0.35, p = 0.08).

## Discussion

In our study, we found that despite safe and effective available vaccination, a large proportion of MSM in the WHO European Region remain vulnerable to both hepatitis A and B, with large variation across countries and reports of low levels of vaccination in many countries. Furthermore, the indirect comparison with earlier analysis of data from EMIS-2010, when 56% of participants reported hepatitis B vaccination history, suggests that the overall hepatitis B vaccination coverage has not increased among MSM in the past years [13]. The COVID-19 pandemic may have additionally negatively affected vaccination coverage among MSM [29]. The presented data have been collected in 2017–18 but rising sexually transmitted infections and evidence of ongoing transmission of hepatitis B in Europe with transmission among MSM as one of the main routes emphasise the relevance of our results and the urgent need for prevention in this key population [7,17,30]. Our results are complemented by the findings of Burdi et al. on viral hepatitis knowledge and immunisation awareness among MSM in Europe [22].

The outbreaks of hepatitis A in recent years among MSM have spread rapidly across European countries and have led to community transmission, given high contagiousness and lack of immunity in the general population [4,31]. Paediatric hepatitis A vaccination was implemented at a national level only in Cyprus, Greece, Israel and Türkiye and at a regional level with catch-up campaigns in Italy (region Apulia) and Spain (regions Catalonia, Ceuta and Melilla) [23]. An overall hepatitis A vaccination coverage of less than 50% among MSM in our study highlights the still existing vaccination gaps across European countries and the need to scale up immunisation and further prevention campaigns to prevent such outbreaks. Based on the transmission dynamics of the 2016–18 hepatitis A outbreaks, it was modelled that a background immunity of at least 70% would be necessary to prevent large hepatitis A outbreaks [32]. For low-endemicity countries, like most countries in Europe, this immunity should be generated through targeted vaccination [3]. According to our short survey, at least four countries (Cyprus, Finland, Poland and Slovenia) introduced MSM-specific hepatitis A vaccination recommendations after the year 2017, potentially leading to increased protection of MSM in these countries.

Since 1992, the WHO has called for the inclusion of hepatitis B vaccination into vaccination schedules for newborns and infants [33]. Additionally, many countries have vaccination programmes targeting adult populations who are at higher risk of hepatitis B infection, such as MSM. At least one additional country (Cyprus) had introduced MSM-specific hepatitis B vaccination recommendation after 2017. Our study showed that on average a higher proportion of MSM were reported to be vaccinated in countries where vaccination was recommended, either free of charge or with co-payment. However, in some countries, vaccination coverage was unexpectedly higher or lower compared to other countries with similar vaccination programmes and levels of outness, indicating that additional factors influence individual vaccination decisions. A mathematical modelling study from the Netherlands, the country with the highest proportion of participants who had received hepatitis B vaccination, showed that stopping vaccination programmes targeting populations at high risk of infection in 2030 and only relying on universal vaccination would result in 30% more new hepatitis B infections until 2070 [34]. The effect of stopping targeted vaccination programmes in other countries would likely be substantial, highlighting the importance of vaccinating key populations at the current stage on the path towards hepatitis elimination.

With the exception of three countries (Denmark, Finland and Iceland), all countries have introduced universal childhood hepatitis B vaccination programmes [27]. In our sample, 21% of participants were in age groups that could have potentially benefitted from these childhood hepatitis B vaccination programmes [28]. However, their vaccination history was unexpectedly lower than that of participants not reached by such programmes. In part, this discrepancy might be attributed to the fact that these programmes had not yet reached their full potential and many infants were missed [21]. Additionally, study participants in younger age groups under 25 years more often lacked awareness of their vaccination status leading to their classification as having no vaccination history, although they might have received vaccination during childhood and did not remember it. This is in line with evidence from a study from the US comparing self-reported vaccination coverage with electronic medical records of adult vaccines and finding lower levels of agreement for both hepatitis A and B vaccinations among younger age groups [35]. Our results suggest that universal childhood vaccination had only a marginal effect on EMIS-2017 participants and underscore the importance of targeted vaccination programmes, as universal programmes need many years to have widespread population effects.

Being out to family and friends increased chances of MSM in our study to have received a hepatitis A or B vaccination. In countries with MSM-specific recommendations, these can only be followed when people are willing to disclose their sexual orientation at least to a healthcare professional. A societal climate that facilitates sexual orientation disclosure is crucial for addressing this key population and targeting prevention measures. Doctors should be informed that vaccines are also recommended for specific groups at high risk of infection and need to be aware of which population groups need targeting for vaccination. In recent decades, the implementation of universal hepatitis B vaccination programmes was facilitated by the development of new vaccines that were effective and safe to use in paediatric care. High vaccination coverage among newborns in the general population will therefore protect future generations from infections with hepatitis B virus regardless of sexuality. Still, our analysis showed that vaccination gaps among adult MSM persist. We argue that information campaigns for MSM, and other groups at high risk, may be helpful to adequately inform healthcare personnel and the public about transmission routes in order to close these gaps [36].

In the multivariable analysis, we found that recommending vaccination for MSM and providing full or partial reimbursement doubled chances of being vaccinated for both hepatitis A and B. When vaccination involved full out-of-pocket payments, we found no association between vaccination recommendation for MSM and vaccination coverage. We observed lower hepatitis A and B vaccination coverage in Bulgaria, Estonia and Iceland than in other countries with similar outness levels. This demonstrates that hepatitis vaccination programmes with out-of-pocket payments did not sufficiently reach MSM and healthcare expenditures may be major barriers for patients when it comes to primary prevention [37]. Austria – the only other country with co-payment for both hepatitis A and B vaccination – had comparatively high coverage, which may be explained by more than 30 years of recommending hepatitis B vaccination to MSM.

In our study, vaccination coverage was generally lower among participants who lived in Eastern European countries and for individuals with migration history from Eastern Europe, Central Asia or the WHO Eastern Mediterranean Region for both hepatitis A and B. In countries where the legal and policy human rights situation of gay, bisexual and trans men is challenging this may hinder access of MSM to healthcare services [38]. Structural stigma towards migrant populations and sexual minorities often leads to poorer access to healthcare services, and a lack of health insurance has been shown to hamper utilisation of prevention services [39]. This is especially true for those who recently migrated or have no official status and can lead to MSM migrants experiencing double stigmatisation. Consideration of recent migration status may offer an opportunity for delivering prevention services but in the long run, only changes in legislation protecting MSM migrants from discrimination based on both immigration status and sexual identity will ensure equitable access to the healthcare system [39].

Participants who had a history of diagnosed HCV infection or HIV in the past were more likely to have received vaccination against hepatitis A and B. Hepatitis B vaccination is generally recommended in clinical guidelines for individuals living with HIV or those with a history of hepatitis C, as liver-related mortality is increased [40,41]. Our findings suggest missed opportunities for prevention and hepatitis A and B vaccinations should be offered to all MSM who are not yet protected through vaccination or immunity following earlier exposure. Individuals should feel encouraged to disclose their sexual behaviour and healthcare professionals should use opportunities to check vaccination records, especially as young MSM may not be aware of their immunity status.

In an accompanying publication, Burdi et al. analysed how EMIS-2017 participants responded to five true statements about viral hepatitis, one of them being the general statement that doctors recommend both hepatitis A and B vaccination for MSM [22]. In our analysis, knowledge about this fact increased slightly among participants with time since a national hepatitis B vaccination recommendation for MSM was implemented, but we found no association for knowledge of this fact and time since introduction of hepatitis A vaccination recommendations for MSM. Knowledge about sexually transmitted infections is crucial, especially for key populations, to protect themselves and make use of preventive measures [22]. In addition to official recommendations and free vaccination for MSM, active promotion of prevention services, proactive healthcare providers, and a societal climate that allows MSM to be open about their sexuality will be essential to increase vaccine uptake.

Our study has some limitations. The EMIS-2017 data are a self-selected, non-random convenience sample and results are not generalisable to all MSM in the WHO European Region. As EMIS-2017 was available exclusively online and promoted mostly through gay dating websites, the sample underrepresents MSM without access to the internet or who do not use online dating websites [18]. Self-reported vaccination status is prone to recall bias and recent evidence from Switzerland suggests that it may overestimate the true proportion of individuals who are actually vaccinated [42]. There may have been misclassification between hepatitis A and B vaccination, when participants entered vaccination information from memory without consulting their vaccination documents. Coding participants who did not know their vaccination status as ‘no vaccination history’ was done to highlight their potential for prevention but may have led to an underestimation of the proportion vaccinated, as some of them may have been vaccinated but not aware. The results of the short focal point surveys on vaccination recommendations are dependent on interpretation of national guidelines and recommendations may have changed over time. In case of unclarities, we consulted with experts and cross-checked with literature but cannot guarantee correct classification of countries at the time of EMIS-2017. Universal hepatitis A vaccination programmes may have reached a few participants in our sample, but we considered this number to be negligible due to the small number of countries and introductions starting in the late 1990s.

## Conclusions

With over 110,000 participants from 43 countries of the WHO European Region, our study offers comprehensive insights into hepatitis A and B vaccination coverage among MSM and associated factors. In countries where vaccination of MSM was recommended and provided for free or with a co-payment, MSM had two to three times higher chances to be protected from hepatitis A and B. Vaccination gaps are still apparent and need to be addressed to protect MSM from hepatitis A outbreaks and long-term consequences of chronic hepatitis B. Burdi et al. also highlight the crucial role of healthcare providers in disseminating information on viral hepatitis and prevention recommendations to key populations by providing an enabling environment. Therefore, implementing national vaccination programmes including free hepatitis A and B vaccination for MSM, raising awareness, reducing stigma, and improving the societal climate for MSM to facilitate access to prevention services are crucial steps in the context of viral hepatitis elimination.

## *Note

This designation is without prejudice to positions on status and is in line with United Nations Security Council Resolution 1244/99 and the International Court of Justice Opinion on the Kosovo Declaration of Independence.

## Supplementary Data

890000 Supplement

## References

[1] Linder KA Malani PN . Hepatitis A. JAMA. 2017;318(23):2393. 10.1001/jama.2017.17244 29094153

[2] Jacobsen KH . Globalization and the changing epidemiology of hepatitis A virus. Cold Spring Harb Perspect Med. 2018;8(10):a031716. 10.1101/cshperspect.a031716 29500305 PMC6169986

[3] World Health Organization . WHO position paper on hepatitis A vaccines - October 2022. Wkly Epidemiol Rec. 2022;97(40):493-512. Available from: https://www.who.int/publications/i/item/who-wer9740-493-512

[4] Ndumbi P Freidl GS Williams CJ Mårdh O Varela C Avellón A Hepatitis A outbreak disproportionately affecting men who have sex with men (MSM) in the European Union and European Economic Area, June 2016 to May 2017. Euro Surveill. 2018;23(33):1700641. 10.2807/1560-7917.ES.2018.23.33.1700641 30131095 PMC6205254

[5] Seto W-K Lo Y-R Pawlotsky J-M Yuen M-F . Chronic hepatitis B virus infection. Lancet. 2018;392(10161):2313-24. 10.1016/S0140-6736(18)31865-8 30496122

[6] World Health Organization (WHO). Hepatitis B Fact Sheet. Geneva: WHO. [Accessed: 18 Jul 2023]. Available from: https://www.who.int/news-room/fact-sheets/detail/hepatitis-b

[7] European Centre for Disease Prevention and Control (ECDC). Hepatitis B - Annual Epidemiological Report for 2022. Stockholm: ECDC; 2024. Available from: https://www.ecdc.europa.eu/sites/default/files/documents/AER%20HEPB%202022_0.pdf

[8] World Health Organization (WHO). Hepatitis B in the WHO European Region. Fact Sheet July 2022. Geneva: WHO; 2022. Available from: https://www.who.int/docs/librariesprovider2/default-document-library/hepatitis-b-in-the-who-european-region-factsheet-july-2022.pdf?sfvrsn=f8eb7bbb_2&download=true

[9] Miglietta A Quinten C Lopalco PL Duffell E . Impact of hepatitis B vaccination on acute hepatitis B epidemiology in European Union/European Economic Area countries, 2006 to 2014. Euro Surveill. 2018;23(6):17-00278. 10.2807/1560-7917.ES.2018.23.6.17-00278 29439751 PMC5824123

[10] Pattyn J Hendrickx G Vorsters A Van Damme P . Hepatitis B vaccines. J Infect Dis. 2021;224(12) Suppl 2;S343-51. 10.1093/infdis/jiaa668 34590138 PMC8482019

[11] Shouval D . Immunization against Hepatitis A. Cold Spring Harb Perspect Med. 2019;9(2):a031682. 10.1101/cshperspect.a031682 29661808 PMC6360863

[12] World Health Organization (WHO). Action plan for the health sector response to viral hepatitis in the WHO European Region. Geneva: WHO; 2017. Available from: www.euro.who.int/en/health-topics/communicable-diseases/hepatitis/publications/2017/action-plan-for-the-health-sector-response-to-viral-hepatitis-in-the-who-european-region-2017

[13] Brandl M Schmidt AJ Marcus U An der Heiden M Dudareva S . Are men who have sex with men in Europe protected from hepatitis B? Epidemiol Infect. 2020;148:e27. 10.1017/S0950268820000163 32052715 PMC7026898

[14] Vet R de Wit JB Das E . Factors associated with hepatitis B vaccination among men who have sex with men: a systematic review of published research. Int J STD AIDS. 2017;28(6):534-42. 10.1177/0956462415613726 26503555

[15] Pachankis JE Hatzenbuehler ML Hickson F Weatherburn P Berg RC Marcus U Hidden from health: structural stigma, sexual orientation concealment, and HIV across 38 countries in the European MSM Internet Survey. AIDS. 2015;29(10):1239-46. 10.1097/QAD.0000000000000724 26035323 PMC4820755

[16] World Health Organization (WHO). Consolidated guidelines on HIV, viral hepatitis and STI prevention, diagnosis, treatment and care for key populations. Report No.: 9240052399. Geneva: WHO; 2022. Available from: https://www.who.int/publications/i/item/9789240052390 36417550

[17] European Commission. Council Recommendation of 21 June 2024 on vaccine-preventable cancers (C/2024/4259). Luxembourg: Publications Office of the European Union. 28.6.2024. Available from: http://data.europa.eu/eli/C/2024/4259/oj

[18] The EMIS Network. EMIS-2017 – The European Men-Who-Have-Sex-With-Men Internet Survey. Key findings from 50 countries. Report No.: 978-92-9498-341-1. Stockholm: European Centre for Disease Prevention and Control; 2019. Available from: https://www.emis-project.eu/european-report-2017

[19] Weatherburn P Hickson F Reid DS Marcus U Schmidt AJ . European men-who-have-sex-with-men internet survey (EMIS-2017): design and methods. Sex Res Soc Policy. 2020;17(4):543-57. 10.1007/s13178-019-00413-0

[20] European Centre for Disease Prevention and Control (ECDC). Vaccine Scheduler. Stockholm: ECDC. [Accessed: 15 Aug 2023]. Available from: https://vaccine-schedule.ecdc.europa.eu

[21] World Health Organization (WHO). Immunization data. Geneva: WHO. [Accessed: 15 Aug 2023]. Available from: https://immunizationdata.who.int/listing.html?topic=&location=

[22] Burdi S Brandl M Marcus U Duffell E Severi E Mozalevskis A Viral hepatitis knowledge and vaccination awareness among men who have sex with men (MSM) in 43 countries of the WHO European Region: results from the European MSM Internet Survey (EMIS-2017). Euro Surveill. 2024; (Forthcoming). 10.2807/1560-7917.ES.2024.29.45.2400099 PMC11544719 39512166

[23] European Centre for Disease Prevention and Control (ECDC). Hepatitis A virus in the EU/EEA, 1975-2014. Stockholm: ECDC; 2016. Available from: https://www.ecdc.europa.eu/en/publications-data/hepatitis-virus-eueea-1975-2014

[24] European Centre for Disease Prevention and Control (ECDC). Surveillance and prevention of hepatitis B and C in Europe. Stockholm: ECDC; 2010. Available from: www.ecdc.europa.eu/en/publications-data/surveillance-and-prevention-hepatitis-b-and-c-europe

[25] European Liver Patients Association. The 2016 Hep-CORE Report. Brussels: ELPA; 2017. Available from: https://elpa.eu/wp-content/uploads/2019/09/Hep-CORE-2016-full-report.pdf

[26] Mereckiene J Cotter S Lopalco P D’Ancona F Levy-Bruhl D Giambi C Hepatitis B immunisation programmes in European Union, Norway and Iceland: where we were in 2009? Vaccine. 2010;28(28):4470-7. 10.1016/j.vaccine.2010.04.037 20451643

[27] Khetsuriani N Mosina L Van Damme P Mozalevskis A Datta S Tohme RA . Progress toward Hepatitis B control - World Health Organization European Region, 2016-2019. MMWR Morb Mortal Wkly Rep. 2021;70(30):1029-35. 10.15585/mmwr.mm7030a1 34324482 PMC8323554

[28] Lernout T Hendrickx G Vorsters A Mosina L Emiroglu N Van Damme P . A cohesive European policy for hepatitis B vaccination, are we there yet? Clin Microbiol Infect. 2014;20(Suppl 5):19-24. 10.1111/1469-0691.12535 24829936

[29] Xiridou M Adam P Meiberg A Visser M Matser A de Wit J The impact of the COVID-19 pandemic on hepatitis B virus vaccination and transmission among men who have sex with men: A mathematical modelling study. Vaccine. 2022;40(33):4889-96. 10.1016/j.vaccine.2022.06.075 35810058 PMC9250904

[30] European Centre for Disease Prevention and Control (ECDC). STI cases on the rise across Europe Stockholm: ECDC; 2024. Available from: https://www.ecdc.europa.eu/en/news-events/sti-cases-rise-across-europe

[31] Friesema IH Sonder GJ Petrignani MW Meiberg AE van Rijckevorsel GG Ruijs WL Spillover of a hepatitis A outbreak among men who have sex with men (MSM) to the general population, the Netherlands, 2017. Euro Surveill. 2018;23(23):1800265. 10.2807/1560-7917.ES.2018.23.23.1800265 29897040 PMC6152169

[32] Zhang X-S Ong JJ Macgregor L Vilaplana TG Heathcock ST Mindlin M Transmission dynamics of the 2016-18 outbreak of hepatitis A among men who have sex with men in England and cost-effectiveness analysis of vaccination strategies to prevent future outbreaks. Lancet Reg Health Eur. 2022;19:100426. 10.1016/j.lanepe.2022.100426 36039276 PMC9417902

[33] World Health Organization (WHO). Global hepatitis report 2017. Report No.: 9241565454. Geneva: WHO; 2017. Available from: https://www.who.int/publications/i/item/9789241565455

[34] Xiridou M Visser M Urbanus A Matser A van Benthem B Veldhuijzen I . Ending risk-group HBV vaccination for MSM after the introduction of universal infant HBV vaccination: A mathematical modelling study. Vaccine. 2021;39(21):2867-75. 10.1016/j.vaccine.2021.04.008 33896665

[35] Rolnick SJ Parker ED Nordin JD Hedblom BD Wei F Kerby T Self-report compared to electronic medical record across eight adult vaccines: do results vary by demographic factors? Vaccine. 2013;31(37):3928-35. 10.1016/j.vaccine.2013.06.041 23806243 PMC4689428

[36] Velan B Yadgar Y . On the implications of desexualizing vaccines against sexually transmitted diseases: health policy challenges in a multicultural society. Isr J Health Policy Res. 2017;6(1):30. 10.1186/s13584-017-0153-4 28666469 PMC5493887

[37] Rezayatmand R Pavlova M Groot W . The impact of out-of-pocket payments on prevention and health-related lifestyle: a systematic literature review. Eur J Public Health. 2013;23(1):74-9. 10.1093/eurpub/cks034 22544911

[38] The European Region of the International Lesbian, Gay, Bisexual, Trans and Intersex Association -Europe. Rainbow Map 2023. Geneva: ILGA; 2023. Available from: https://www.ilga-europe.org/files/uploads/2023/05/rainbow-map-2023.pdf

[39] Pachankis JE Hatzenbuehler ML Berg RC Fernández-Dávila P Mirandola M Marcus U Anti-LGBT and anti-immigrant structural stigma: an intersectional analysis of sexual minority men’s HIV risk when migrating to or within Europe. J Acquir Immune Defic Syndr. 2017;76(4):356-66. 10.1097/QAI.0000000000001519 28787329 PMC5659919

[40] Maqsood Q Sumrin A Iqbal M Younas S Hussain N Mahnoor M Hepatitis C virus/hepatitis B virus coinfection: Current prospectives. Antivir Ther. 2023;28(4):13596535231189643. 10.1177/13596535231189643 37489502

[41] Hu J Liu K Luo J . HIV-HBV and HIV-HCV coinfection and liver cancer development. Cancer Treat Res. 2019;177:231-50. 10.1007/978-3-030-03502-0_9 30523627

[42] Schmidt AJ Rasi M Esson C Christinet V Ritzler M Lung T The Swiss STAR trial - an evaluation of target groups for sexually transmitted infection screening in the sub-sample of men. Swiss Med Wkly. 2020;150(5153):w20392. 10.4414/smw.2020.20392 33382077

