# Supplementary material for: Self-reported hepatitis A and B vaccination coverage among men who have sex with men (MSM), associated factors and vaccination recommendations in 43 countries of the WHO European Region: results from the European MSM Internet Survey, EMIS-2017
Source: Euro Surveill. 2024 Nov 7;29(45):2400100. doi: 10.2807/1560-7917.ES.2024.29.45.2400100 (PMC11544724; doi:10.2807/1560-7917.ES.2024.29.45.2400100)
Supplement: Supplement [file 24-00100_BRANDL_Supplement.pdf]

Supplement

This supplementary material is hosted by *Eurosurveillance* as supporting information alongside the article "self-reported hepatitis A and B vaccination coverage among men who have sex with men (MSM), associated factors, and vaccination recommendations in 43 countries of the WHO European Region: results from the European MSM Internet Survey, EMIS-2017", on behalf of the authors, who remain responsible for the accuracy and appropriateness of the content. The same standards for ethics, copyright, attributions and permissions as for the article apply. Supplements are not edited by *Eurosurveillance* and the journal is not responsible for the maintenance of any links or email addresses provided therein.

Supplementary Table S1. Numbers and percentages of participants with hepatitis A and B vaccination history by country of residence

Country of residence	Hepatitis A vaccination history			Hepatitis B vaccination history		
	n	%	Total	n	%	Total
Albania/Kosovo/Montenegro	33	27	121	38	31	124
Austria	1,550	66	2,364	1,554	66	2,358
Belarus	48	13	373	51	14	371
Belgium	1,540	60	2,548	1,649	64	2,561
Bosnia & Herzegovina	52	28	189	61	32	189
Bulgaria	147	16	906	171	19	902
Croatia	306	37	834	412	49	843
Cyprus	104	40	258	125	49	257
Czechia	750	47	1,611	714	44	1,610
Denmark	791	54	1,458	792	55	1,444
Estonia	58	32	179	61	34	181
Finland	712	56	1,277	640	50	1,274
France	4,021	46	8,704	5,442	60	9,099
Germany	13,035	65	20,126	12,949	64	20,103
Greece	1,075	46	2,347	1,186	51	2,341
Hungary	522	29	1,787	567	32	1,785
Iceland	49	49	99	47	49	96
Ireland	975	55	1,784	1,107	62	1,793
Israel	496	49	1,008	492	48	1,033
Italy	2,606	33	8,016	3,814	46	8,298
Latvia	63	30	210	60	29	206
Lithuania	54	20	271	66	24	274
Luxembourg	91	65	141	100	68	147
Malta	136	53	255	147	57	258
Moldova	125	30	418	165	38	439
Netherlands	2,084	65	3,209	2,433	77	3,167
North Macedonia	13	10	133	29	21	137
Norway	1,337	51	2,644	1,463	55	2,646
Poland	1,056	32	3,302	1,358	40	3,414
Portugal	901	44	2,047	1,134	53	2,135
Romania	343	23	1,523	383	25	1,520
Russia	996	20	4,959	1,264	25	5,151
Serbia	160	20	798	221	28	793
Slovakia	302	35	855	284	33	848
Slovenia	212	37	580	246	42	582
Spain	4,057	46	8,734	4,392	49	8,911
Sweden	1,926	50	3,819	1,855	49	3,790
Switzerland	1,930	67	2,891	2,022	70	2,898
Turkey	432	34	1,264	563	43	1,301
Ukraine	74	8	939	120	13	944
United Kingdom	5,804	56	10,274	6,712	65	10,330

Supplementary Table S2. Descriptive comparison of participants who stated they were not vaccinated and participants who did not know their vaccination status

Variables	Hepatitis A vaccination				Hepatitis B vaccination			
	Not vaccinated n = 30,092		Unknown n = 24,197		Not vaccinated n = 26,294		Unknown n = 23,370	
	n	%	n	%	n	%	n	%
**Age group in years**								
< 25	6,069	20	7,344	30	5,473	21	7,230	31
25–39	12,751	42	10,862	45	10,602	40	10,415	45
≥ 40	11,272	37	5,991	25	10,219	39	5,725	24
**Settlement size**								
Medium-sized or smaller (< 500,000 inhabitants)	17,216	58	13,928	58	15,191	58	13,333	58
Big to very big cities (≥ 500,000 inhabitants)	12,551	42	9,991	42	10,809	42	9,765	42
**Education**								
Low (0–1 year post age 16 years)	1,579	6	1,366	6	1,415	6	1,308	6
Mid (at least upper secondary; 2–5 years post age 16 years)	10,492	38	9,443	42	9,693	40	9,194	42
High (first stage of tertiary or more; ≥ 6 years post age 16 years)	15,802	57	11,698	52	13,206	54	11,218	52
**Financial coping**								
Struggling/really struggling with present income	5,563	19	4,970	21	4,839	18	4,851	21
Neither comfortable nor struggling with present income	10,993	37	9,396	39	9,666	37	9,018	39
Living comfortably/really comfortably with present income	13,418	45	9,691	40	11,671	45	9,366	40
**Outness**								
Out to none or few	10,979	37	9,060	38	10,049	39	8,873	39
Out to some	8,872	30	7,018	29	7,495	29	6,752	29
Out to (almost) all	9,820	33	7,740	32	8,360	32	7,362	32
**Ever diagnosed with hepatitis C or HIV**								
No	1,877	6	1,274	5	1,461	6	1,142	5
Yes	28,045	94	22,811	95	24,682	94	22,119	95
**Country of birth**								
No migration history	26,798	89	21,209	88	23,404	89	20,494	88
EU/EEA, Switzerland, or UK	1,567	5	1,454	6	1,331	5	1,381	6
Other countries of the WHO European Region	613	2	482	2	558	2	482	2
Who eastern mediterranean region	166	0.6	169	0.7	144	0.5	148	0.6
Australia, Canada, New Zealand, or US	138	0.5	122	0.5	123	0.5	111	0.5
All other countries	729	2	694	3	658	3	685	3
**MSM-specific hepatitis A recommendation**								
No recommendation	9,973	33	7,834	32	NA			
Out-of-pocket	1,161	4	800	3				
Co-payment	6,102	20	4,640	19				
Free of charge	12,856	43	10,923	45				
**MSM-specific hepatitis B recommendation**								
No recommendation	NA				4,980	19	4,168	18
Out-of-pocket					2,694	10	1,937	8
Co-payment					4,652	18	4,007	17
Free of charge					13,923	53	13,217	57
**Universal hepatitis B programme**								
Not reached	NA				21,294	81	15,608	67
Age groups potentially reached					5,000	19	7,762	33

EMIS-2017: European MSM Internet survey; EU/EEA: European Union/European Economic Area; HIV: human immunodeficiency virus: MSM: men who have sex with men; NA: not applicable; UK: United Kingdom; US: United States; WHO: World Health Organization.

Supplementary Table S3: Sensitivity analysis of multivariable regression excluding participants not knowing their hepatitis A and B vaccination status

Variables	Hepatitis A vaccination history		Hepatitis B vaccination history	
	aOR	95% CI	aOR	95% CI
**Age in years**				
< 25	0.86	0.82–0.90	0.70	0.66–0.74
25–39	Ref.		Ref.	
≥ 40	0.79	0.76–0.82	0.75	0.72–0.78
**Settlement size**				
Medium-sized or smaller (< 500 000 inhabitants)	Ref.		Ref.	
Big to very big cities (≥ 500 000 inhabitants)	1.36	1.31–1.40	1.34	1.30–1.39
**Education**				
Low (0–1 year post age 16 years)	Ref.		Ref.	
Mid (at least upper secondary; 2–5 years post age 16 years)	1.29	1.19–1.40	1.28	1.18–1.38
High (first stage of tertiary or more; ≥ 6 years post age 16 years)	1.60	1.48–1.73	1.87	1.73–2.03
**Financial coping**				
Struggling/really struggling with present income	Ref.		Ref.	
Neither comfortable nor struggling with present income	1.20	1.14–1.26	1.18	1.13–1.24
Living comfortably/really comfortably with present income	1.61	1.54–1.69	1.56	1.49–1.64
**Outness**				
Out to none or few	Ref.		Ref.	
Out to some	1.31	1.25–1.37	1.40	1.34–1.46
Out to (almost) all	1.75	1.67–1.82	1.79	1.72–1.87
**Ever diagnosed with hepatitis C or HIV**				
No	Ref.		Ref.	
Yes	2.67	2.51–2.84	2.97	2.78–3.17
**Country of birth**				
No migration history	Ref.		Ref.	
EU/EEA, Switzerland, or UK	1.16	1.09–1.25	1.17	1.09–1.26
Other countries of the WHO European Region	0.68	0.59–0.77	0.71	0.62–0.81
WHO Eastern Mediterranean Region	0.80	0.64–1.00	0.84	0.67–1.05
Australia, Canada, New Zealand, or US	1.76	1.43–2.17	1.57	1.26–1.95
All other countries	1.16	1.05–1.28	1.03	0.93–1.13
**MSM-specific hepatitis A vaccination recommendation**				
No recommendation	Ref.		NA	
Out-of-pocket	1.03	0.50–2.13		
Co-payment	2.33	1.25–4.34		
Free of charge	2.20	1.18–4.12		
**MSM-specific hepatitis B vaccination recommendation**				
No recommendation	NA		Ref.	
Out-of-pocket			1.57	0.84–2.94
Co-payment			3.16	1.62–6.18
Free of charge			2.66	1.57–4.51
**Universal hepatitis B vaccination programme**				
Not reached	NA		Ref.	
Age groups potentially reached			1.50	1.42–1.59

aOR: adjusted odds ratio; CI: confidence interval; EU/EEA: European Union/European Economic Area; HIV: human immunodeficiency virus; MSM = men who have sex with men; NA: not applicable; UK: United Kingdom; US: United States; WHO: World Health Organization.

Supplementary Table S4: Sensitivity analysis excluding age groups which were potentially reached by universal hepatitis B vaccination programmes

**Variables**	**Hepatitis B vaccination history**	
	**aOR**	**95% CI**
**Age in years**		
< 25	0.73	0.69–0.77
25–39	Ref.	
≥ 40	0.91	0.88–0.95
**Settlement size**		
Medium-sized or smaller (< 500 000 inhabitants)	Ref.	
Big to very big cities (≥ 500 000 inhabitants)	1.34	1.30–1.39
**Education**		
Low (0–1 year post age 16 years)	Ref.	
Mid (at least upper secondary; 2–5 years post age 16 years)	1.26	1.17–1.36
High (first stage of tertiary or more; ≥ 6 years post age 16 years)	1.83	1.70–1.98
**Financial coping**		
Struggling/really struggling with present income	Ref.	
Neither comfortable nor struggling with present income	1.25	1.19–1.31
Living comfortably/really comfortably with present income	1.71	1.63–1.79
**Outness**		
Out to none or few	Ref.	
Out to some	1.52	1.45–1.58
Out to (almost) all	1.96	1.89–2.04
**Ever diagnosed with hepatitis C or HIV**		
No	Ref.	
Yes	2.93	2.77–3.11
**Country of birth**		
No migration history	Ref.	
EU/EEA, Switzerland, or UK	1.04	0.98–1.11
Other countries of the WHO European Region	0.70	0.62–0.80
WHO Eastern Mediterranean Region	0.86	0.70–1.06
Australia, Canada, New Zealand, or US	1.31	1.10–1.56
All other countries	0.97	0.88–1.07
**MSM-specific hepatitis B vaccination recommendation**		
No recommendation	Ref.	
Out-of-pocket	1.74	1.03–2.96
Co-payment	3.04	1.73–5.36
Free of charge	2.50	1.60–3.90

aOR: adjusted odds ratio; CI: confidence interval; EU/EEA: European Union/European Economic Area; HIV: human immunodeficiency virus; MSM = men who have sex with men; NA: not applicable; UK: United Kingdom; US: United States; WHO: World Health Organization.

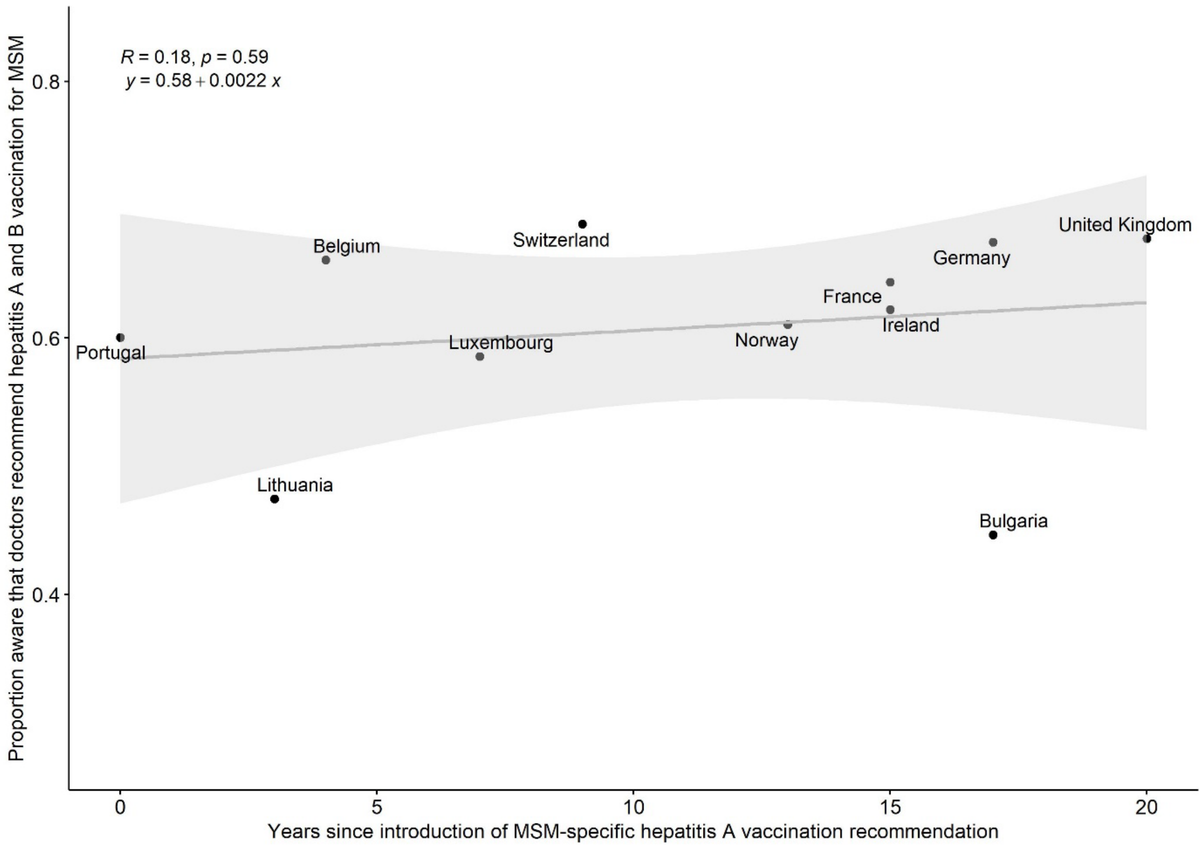

Supplementary Figure S1: Correlation between the time since introduction of MSM-specific hepatitis A vaccination recommendation and the proportion of participants per country aware of doctors recommending MSM to be vaccinated against hepatitis A and B viruses (n = 11)

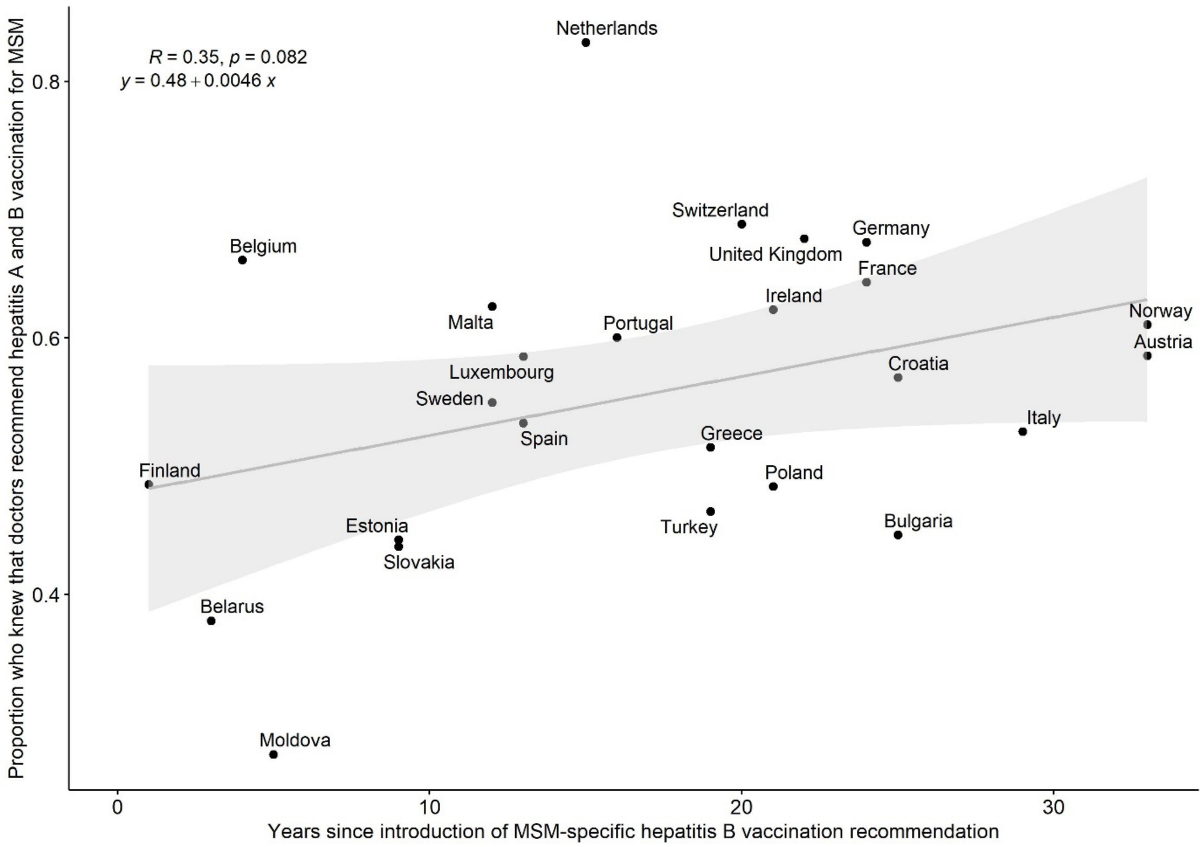

Supplementary Figure S2: Correlation between the time since introduction of MSM-specific hepatitis A vaccination recommendation and the proportion of participants per country aware of doctors recommending MSM to be vaccinated against hepatitis A and B viruses (n = 25, results for Albania not shown)
